# Butyrate facilitates immune clearance of colorectal cancer cells by suppressing STAT1-mediated PD-L1 expression

**DOI:** 10.1016/j.clinsp.2023.100303

**Published:** 2023-11-05

**Authors:** Yan Zhang, Yuan Tao, Yuqing Gu, Qiujie Ma

**Affiliations:** a Department of Pathology, Beijing Changping Traditional Chinese Medicine Hospital, Beijing, China; b Department of Gastroenterology, Beijing Changping Traditional Chinese Medicine Hospital, Beijing, China; c Department of Pathology, Guang'anmen Hospital South Campus, China Academy of Chinese Medical Sciences, Beijing, China

**Keywords:** Colorectal Cancer, Immunotherapy, Butyrate, PD-L1, STAT1

## Abstract

**Objective:**

Immunotherapy has been proven to improve the prognosis of patients with advanced malignancy but has shown limited efficacy in patients with Colorectal Cancer (CRC). Increasing evidence suggests that butyrate, a bacterial metabolite, enhances the efficacy of cancer therapies by modulating immune responses. Here, the effect and the mechanism of butyrate on anti-PD-L1 therapy were investigated in CRC.

**Methods:**

The expression of PD-L1 and STAT1, and the lysine acetylation of STAT1 in CRC cells were observed after treatment with butyrate (2, 5, and 10 mM) for 24h or butyrate (5 mM) for 8, 16, and 24h. Site-directed mutations of STAT1 (K410R or K413R) were introduced to determine the role of STAT1 acetylation in modulating PD-L1 expression. The effect of butyrate on the cytotoxicity of CD8+ T-cells against CRC cells with or without PD-L1 overexpression was explored in vitro and in vivo.

**Results:**

Butyrate could suppress IFN-γ-induced PD-L1 up-regulation in CRC cells in a dose- and time-dependent way. Butyrate promoted the lysine acetylation of STAT1 to reduce STAT1 expression. Non-acetylated mutant STAT1 not only ameliorated butyrate-induced suppression of lysine acetylation and nuclear translocation of STAT1 but also blocked the effect of butyrate on PD-L1. Butyrate attenuated the IFN-γ-induced impairment of CD8+ T-cell cytotoxicity against CRC cells. Meanwhile, butyrate suppressed CRC tumor growth by enhancing CD8+ T-cell infiltration. However, directly overexpressing PD-L1 in CRC cells could abolish the effect of butyrate.

**Conclusion:**

Butyrate strengthens the immune response to CRC cells by suppressing PD-L1 expression via acetylation of STAT1.

## Highlights

• Butyrate could suppress IFN-γ-induced PD-L1 up-regulation in Colorectal Cancer (CRC) cells.
• Butyrate promoted the lysine acetylation of STAT1 to reduce STAT1 expression.
• Butyrate attenuated the IFN-γ-induced impairment of CD8+ T-cell cytotoxicity against CRC cells.
• Butyrate suppressed CRC tumor growth by enhancing CD8+ T-cell infiltration.

## Introduction

As reported, there are approximately 1.15 million new Colorectal Cancer (CRC) cases, representing 5.5% of mortality, in 2020 around the world.^1^ As the fifth most common malignancy, CRC remains a lethal public health issue even though the advances in detection and treatment achieved in the past three decades.^2^ At the early stages of CRC, the common therapy is radiotherapy and/or chemotherapy following surgical resection;^3^ but these treatments may lead to some obstacles including their adverse effects, and the development of therapeutic resistance.^4^ Hence, to overcome these challenges, other therapeutic strategies are urgently required to be explored.

Among several developing therapeutic approaches, immuno-oncology, also called cancer immunotherapy, is becoming the considerably promising option in cancer therapy, which has been successfully exploited for the therapeutic strategy of multiple malignancies, such as renal cell carcinoma,^5^ non-small cell lung cancer,^6^ and CRC.^7^ Cancer immunotherapy aims to boost a self-sustaining cancer-immunity cycle to battle malignant tumors. Immune checkpoint molecules and the corresponding receptors, such as CTLA4, PD-1, and PD-L1, exert pivotal functions in modulating the immune system, of which overexpression in malignant cells could help them escape from immune attack.^8^ Therefore, directly targeting immune checkpoints by antibodies could be considered to be a promising therapy for patients with a variety of malignancies.

Nowadays, antibodies blocking PD-1/PD-L1 exhibit exceptional clinical response for some patients and have been approved to be a standard-of-care treatment in a still-increasing number of types of malignancies.^9^, ^10^, ^11^ However, the existence of resistance impedes the clinical practice of anti-PD-1 therapy; for example, only approximately 20%–30% of patients have positively responded to anti-PD-1/PD-L1 therapy while only a minority of them could achieve a complete response.^12^^,^^13^ Accordingly, significant efforts to identify novel effective biomarkers and mechanisms underlying resistance against anti-PD-1/PD-L1 therapy are currently being undertaken to amplify its efficacy and clinical response.

During tumorigenesis, in the tumor microenvironment, Signal Transducers and Activators of Transcription (STAT) determine the relation between the host immune system and tumor cells.^14^ Accumulating reports demonstrated that the phosphorylated-STAT1 (p-STAT1) is a prospective biomarker for investigating anti-PD‑1/PD‑L1 immunotherapy, which could up-regulate PD-L1 expression following IFN-γ stimulation in several types of tumors, highlighting the importance of STAT1 for PD-L1 expression.^15^, ^16^, ^17^ Butyrate, a short-chain fatty acid, is mainly produced by intestinal microbiota, which has been proven to effectively prevent CRC.^18^^,^^19^ Most data support that the acetylation of STAT1 leads to a switch to inactivate STAT1.^20^ Notably, a previous study reported that butyrate functioned as a Histone Deacetylase (HDAC) inhibitor and suppressed IFN-γ-induced STAT1 activation.^21^ In this context, it would be interesting to explore whether butyrate can also be involved in the absence of STAT1 activation partially helping to inhibit PD-L1 expression in CRC, thereby enhancing the cytotoxicity of T-cells to tumor cells.

The present study first explored the effect and the mechanism of butyrate on the PD-L1 expression of CRC cells, with the aim of providing a potential strategy against immune escape in clinical synergy immunotherapy.

## Materials and methods

### Cell culture and treatment

Three CRC cell lines including HCT116, Lovo, and MC38, were obtained from ATCC (VA, USA) and iCell Bioscience Inc. (Shanghai, China), and were grown in RPMI 1640 medium (10% FBS and 1% penicillin-streptomycin). Cells were cultivated in a humidified environment of 5% CO_2_ at 37°C.

TALL-104 (ATCC), CD8+ cytotoxic T-cells, were cultured in ATCC-formulated IMDM containing IL-2 (100 U/mL), albumin (2.5 μg/mL), D-mannitol (0.5 μg/mL), 20% FBS and 1% penicillin-streptomycin.

IFN-γ (20 ng/mL) was exploited to induce the expression of PD-L1 in CRC cells. Meanwhile, cells were exposed to a series of concentrations (2, 5, and 10 mM) of butyrate for 8, 16, and 24h.

For the detection of cytotoxicity of CD8+ T-cells to CRC cells, tumor cell-immune cell co-culture was performed. In brief, Adherent HCT116 cells were co-cultured for 16h with TALL-104 cells at a ratio of 1:1. HCT116 cells were incubated with IFN-γ alone or combined with butyrate for 24h prior to adding TALL-104 cells. Upon completion of 16h co-culture, the culture medium was removed together with suspended TALL-104 cells.

### Cell transfection

To achieve the overexpression of PD-L1, the PD-L1 gene fragments (NM_014143.2) amplified by PCR were subcloned into the pcDNA3.1 vector (Invitrogen, USA), which subsequently transfected into CRC cells using Lipofectamine 2000 using Lipofectamine® 3,000 reagent (Invitrogen, USA). The empty vector was used as a negative control.

STAT1 was knocked down using specific siRNA (si-STAT1, 5’-CCCUAGAAGACUUACAAGAUGAAUA-3’) with the transfection reagent. After silencing STAT1, unacetylated mutation on the K410 and K413 on STAT1 was carried out based on Flag-tagged expression vectors (Sigma-Aldrich, USA) with QuickMutation™gene mutagenesis kit (Beyotime, China) in accordance with manufacturer's instructions.

### Western lot (WB)

The total protein was isolated from tissues and cells with RIPA buffer (10×) (Cell Signaling, USA) and then subjected to quantification with a BCA assay kit (Sigma-Aldrich, USA). The total protein was separated by SDS-PAGE gel and subsequently transferred onto PVDF membranes. After blocking with skim milk, membranes were incubated with primary antibodies specific for PD-L1 (Abcam, USA; #ab205921), STAT1 (Thermo Fisher, USA; #AHO0832), p-STAT1 (Invitrogen, USA; #PA5-97355), acetyl Lysine (Ac-Lys) (Abcam; #ab90479), and GADPH (Beyotime, China; #AF0006) overnight. Next, membranes were washed thrice prior to 2h incubation with HRP-conjugated secondary antibody. At last, an ECL detection reagent (Sigma-Aldrich) was used to visualize protein bands.

### Co-immunoprecipitation (Co-IP)

HCT116 cells were lysed and incubated with anti-STAT1 antibody (IgG was used as negative control), or bead-conjugated FLAG overnight at 4°C. Then, lysis buffer was utilized to rinse beads coupling immuno-complexes prior to eluting precipitated proteins with SDS-PAGE buffer. Finally, the separation of eluted proteins was performed with SDS-PAGE gels. The interacting proteins were subjected to WB for detection of STAT1 and Ac-Lys.

### Immunofluorescence (IF)

After treatment with IFN-γ combined with butyrate (0 or 5 mM) for 24h, HCT116 cells with diverse transfections seeded onto cell culture dishes were washed and fixed. Then, the cells were permeabilized for 10 min and blocked for 1h before incubation with anti-Flag antibodies (#ab18230; Abcam, USA) overnight. Next, the samples were washed and incubated with secondary antibodies for 1h. Finally, the samples were washed thrice, of which the nuclei were stained with DAPI for 10 min prior to observation with confocal microscopy.

### Luciferase reporter assay

Briefly, STAT1-null HCT116 cells were transiently transfected with the FLAG-STAT1 K410R K413R plasmid or FLAG-STAT1 WT control. The Renilla luciferase plasmid pRL-TK served as an internal control. After 24h, 5 mM of butyrate was added for an additional 24h incubation. The Dual-Luciferase Reporter Assay System (Promega, USA) was exploited to assess the luciferase activities of each group.

### Chromatin immunoprecipitation-quantitative real-time PCR (ChIP-qPCR) assay

After being transiently transfected with the FLAG-STAT1 K410R, FLAG-STAT1 K413R plasmids, or FLAG-STAT1 WT control, STAT1-null HCT116 cells were incubated with IFN-γ combined with PBS as control and 5 mM butyrate. Twenty-four later, the nuclear lysate was collected to make small DNA fragments of approximately 150–900 base pairs with sonication. Then, the small DNA fragments were incubated with ChIP grade anti-Flag antibody overnight. The immunocomplexes were harvested with protein A/G agarose beads. Finally, after DNA elution, 5% of respective input DNA was reverse cross-linked at 65°C overnight for the subsequent qPCR analysis. ChIP-enriched chromatin was used for qPCR with SYBR green reagent on the 7500 Real-Time PCR System (Applied Biosystems, USA). The primers of sequences for PD-L1 are as follows: Forward: 5’-GCTTTTCAATGTGACCAGCA-3’, reverse 5’-TGGCTCCCAGAATTACCAAG-3’.

### Cell viability analysis

To observe the viability of HCT116 cells, a CCK-8 assay was carried out after cells in 96-well plates finished the treatment. 10% of CCK-8 reagent (Dojindo) was added into each well and cultivated for another 2h before the plate was subjected to detecting the Optical Density (OD) of each well at 450 nm with a microplate reader (Bio-Rad, USA).

### Cell apoptosis detection

FITC-Annexin V/PI staining assay was applied to detect the apoptosis of HCT116 cells with diverse interventions. In brief, treated cells were harvested and rinsed twice with PBS. Next, the Annexin-V dye solution (5 μL) and propidium iodide dye solution (10 μL) from the assay kit (Sigma-Aldrich, USA) were added to stain the dark at 4°C. After staining for 30 min, the cells were collected for checking the apoptosis rate using Flow Cytometry (FCM).

### In vivo animal studies

All animal procedures were approved by the Institutional Animal Care and Use Committee at Guang'anmen Hospital South Campus, China Academy of Chinese Medical Sciences. A total of 36 BALB/c male mice (18‒22g, 6‒8 weeks) purchased from Beijing Vital River Laboratory Animal Technology Co., Ltd.. were maintained under SPF conditions and subjected to one-week acclimation prior to the following experiments.

Initially, PD-L1 overexpressing MC38 cells or MC38 cells control were subcutaneously injected into mice with a density of 800,000 cells. To determine the effect of butyrate on colorectal tumor growth, mice were randomly divided into six groups (n = 6 per group): MC38+control, MC38+vehicle, MC38+butyrate, MC38+LV-PD-L1+control, MC38+LV-PD-L1+vehicle, MC38+LV-PD-L1+butyrate. Intra-tumoral administration is performed according to the group (twice a week) when the tumor reaches 50 mm^3^. On day 7, the length and width of tumors were monitored with a ruler once a week for four weeks and the tumor volume was calculated using the following equation: Tumor volume = (length × width^2^)/2, and the growth curve of tumors was plotted. At the end of the experiment (Day 21), the mice were sacrificed by cervical dislocation and the tumors were collected for weight and subsequent analyses.

### Immunohistochemistry (IHC)

The collected tumors were fixed in 4% paraformaldehyde and then embedded in paraffin. Paraffin-embedded tumor sections from different tumors were cut into5 µm slides and subsequently incubated with anti-CD8 antibodies overnight. The following day, the sections were incubated with the secondary antibodies before staining the nuclei with hematoxylin. Finally, the slices were dehydrated, cleaned and mounted.

### Tumor-infiltrating lymphocyte staining

To prepare single-cell suspensions, the half of tumors excised on Day 21 were incubated with collagenase type IV and DNAase (0.02 mg/mL) for 30 min and minced through a cell strainer (70-μm). After washing with RPMI-1640 and FACS buffer, live cells were superficially stained with antibodies specific to CD3 and CD8 at 4°C for 30 min. Flow cytometry analysis was conducted using a FACSAria flow cytometer and analyzed with FlowJo software.

### Statistical analysis

All data were expressed as mean± standard error of the mean. The Student's t-test was used for comparison between the two groups. For multiple-group conditions, one-way ANOVA was performed with Bonferroni's method; p < 0.05 was considered statistically significant.

## Results

### Butyrate suppresses IFN-γ-induced PD-L1 expression in CRC cells

**Fig. 1:**
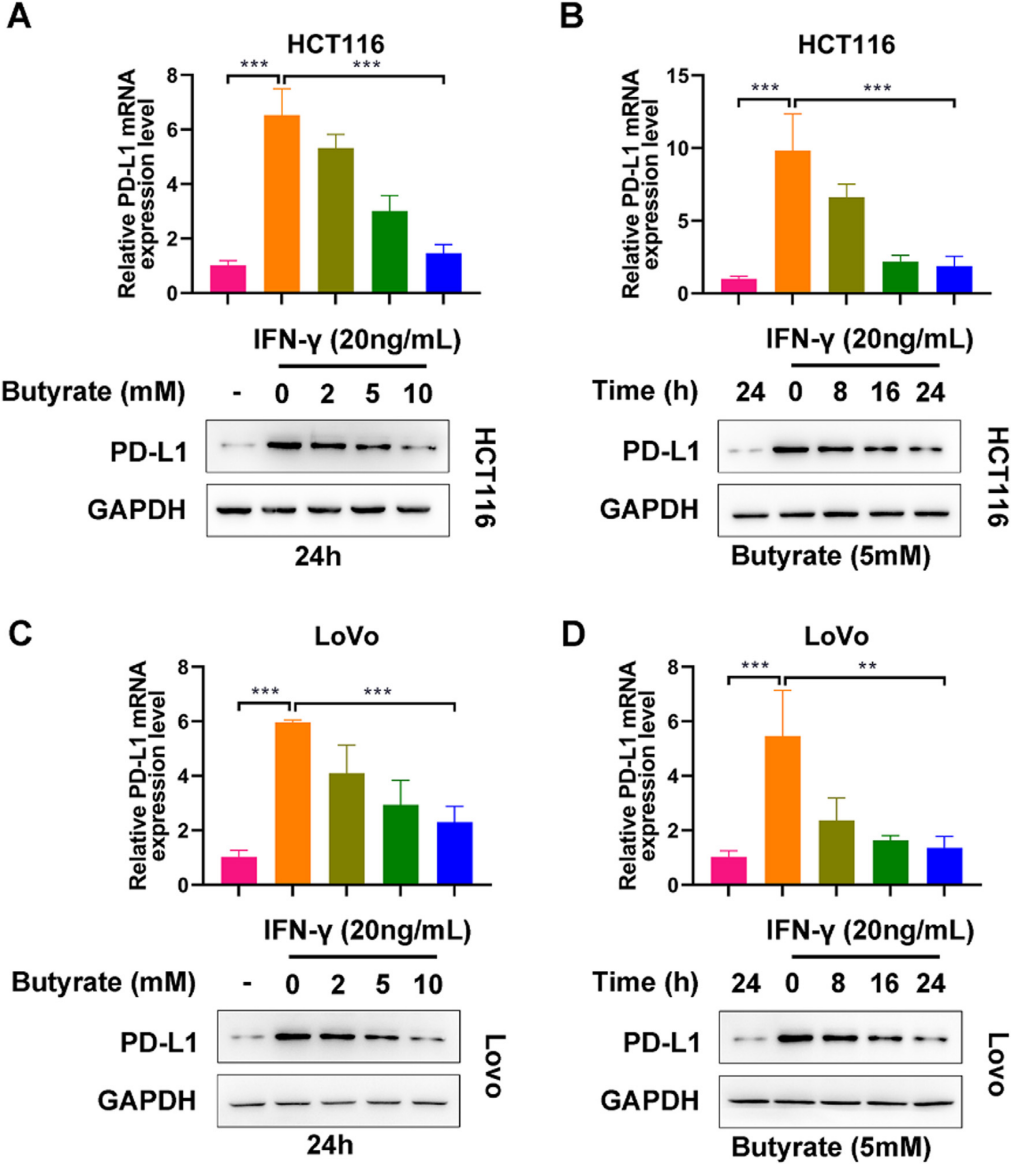
Butyrate suppresses IFN-γ-induced PD-L1 expression in CRC cells. (A) The protein expression of PD-L1 in IFN-γ-induced HCT116 cells after treatment with different concentrations of butyrate for 24h. (B) The protein expression of PD-L1 in IFN-γ-induced HCT116 cells after treatment with 5 mM of butyrate for different incubation times. (C) The protein expression of PD-L1 in IFN-γ-induced LoVo cells after treatment with different concentrations of butyrate for 24h. (D) The protein expression of PD-L1 in IFN-γ-induced LoVo cells after treatment with 5 mM of butyrate for different incubation times. ***p < 0.005.

To observe the modulatory role of butyrate in PD-L1 expression of IFN-γ-treated CRC cells, HCT116 and LoVo cells were exposed to butyrate (0, 2, 5, or 10 mM for 24h or 5 mM for 0, 8, 16 or 24h) under the interference of 24h treatment with IFN-γ (20 ng/mL). As expected, without the administration of butyrate, IFN-γ caused a significant elevation in PD-L1 expression levels in both HCT116 and LoVo cells (Fig 1 A‒D). In HCT116 cells, it could be observed that the up-regulation induced by IFN-γ was blocked by butyrate in the dose- and time-dependent way (Fig 1 A and B). Similar results were found in LoVo cells (Fig 1 C and D). Collectively, butyrate could suppress IFN-γ-induced PD-L1 expression in CRC cells.

### Butyrate suppresses the PD-L1 expression by enhancing STAT1 acetylation in CRC cells

**Fig. 2:**
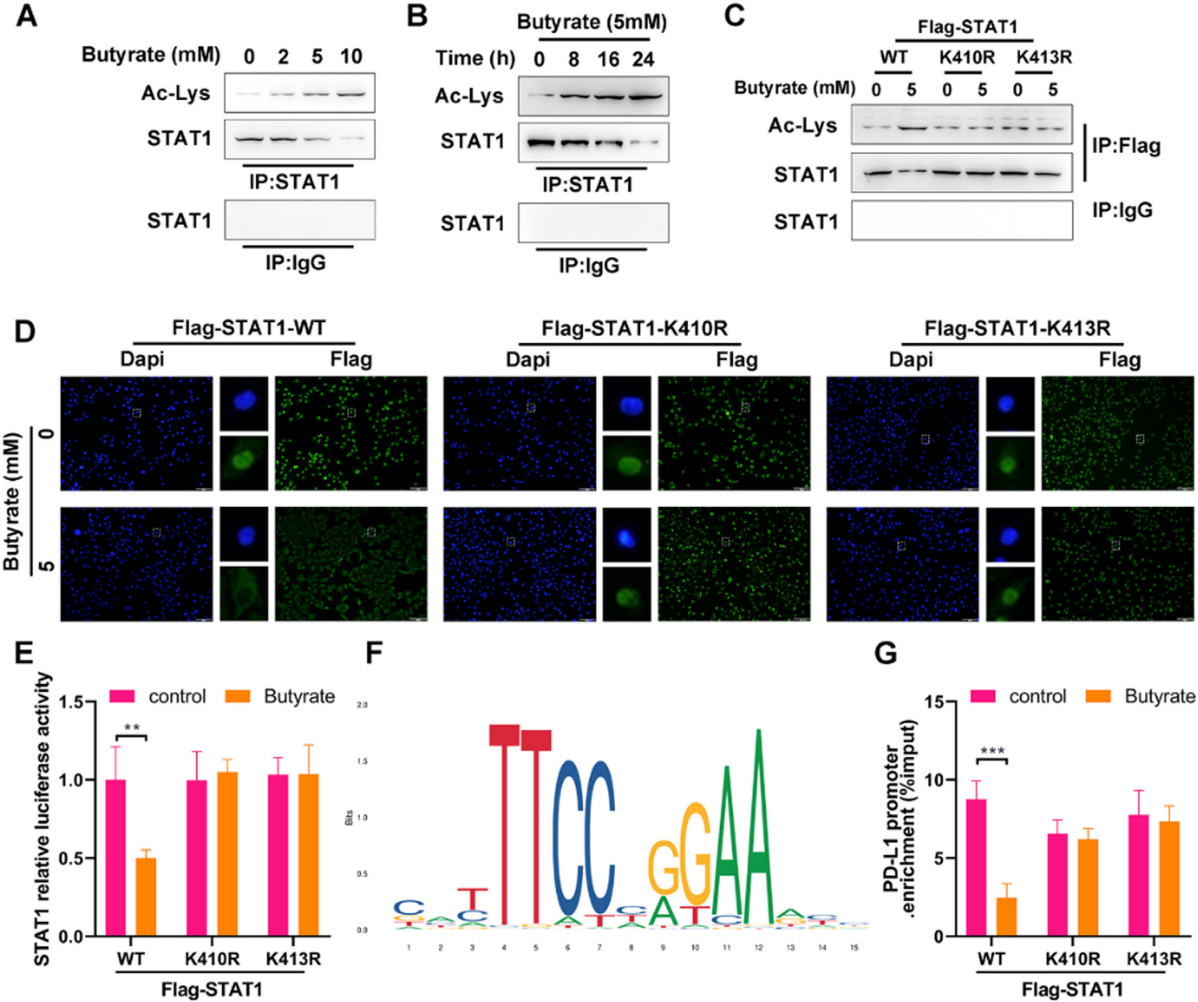
Butyrate suppresses the PD-L1 expression by enhancing STAT1 acetylation in CRC cells. (A) The protein expression of Ac-Lys and STAT1 in anti-STAT1-precipitated complexes of IFN-γ-induced HCT116 cells after treatment with different concentrations of butyrate for 24h. Anti-IgG served as a negative control. (B) The protein expression of Ac-Lys and STAT1 in anti-STAT1-precipitated complexes of IFN-γ-induced HCT116 cells after treatment with 5 mM of butyrate for different incubation times. Anti-IgG served as a negative control. Before treatment with IFN-γ combined with or without 5 mM of butyrate, HCT116 cells were transfected with FLAG-STAT1 WT, FLAG-STAT1 K410R, or FLAG-STAT1 K413R plasmids. (C) The protein expression of Ac-Lys and STAT1 in anti-Flag-precipitated complexes of HCT116 cells. (D) IF staining for STAT1 in HCT116 cells. (E) Luciferase reporter assay for STAT1 transcription activity in HCT116 cells. (F) DNA motif of STAT1 obtained from JASPAR. (G) ChIP-qPCR for PD-L1 promoter in anti-Flag-precipitated complexes of HCT116 cells. **p < 0.01 and ***p < 0.005.

As the essential role of STAT1 activation in PD-L1 up-regulation, the effect of butyrate on STAT1 was investigated. As shown in Figs 2 A and B, the Co-IP analysis displayed that butyrate increased STAT1 acetylation in the dose- and time-dependent way, as confirmed by the Ac-Lys levels in STAT1 immunoprecipitate fractions of HCT116 cells. To inspect whether the STAT1 unacetylate mutation could resist acetylation with the presence of butyrate, HCT116 cells were co-transfected with si-STAT1 and either FLAG-STAT1 K410R/K413R plasmids or FLAG-STAT1 WT control. The results showed that STAT1 mutant at K410R and K413R was more resistant to butyrate-induced acetylation than STAT1 WT (Fig 2C), which suggested that acetylation of STAT1 occurs at the K410 and K413 molecular sites.

Since STAT1 activation exerts the function following the redistribution of STAT1 from the cytoplasm to the nucleus, the effect of STAT1 acetylation on the butyrate-induced suppression of STAT1 nuclear translocation was further verified. Under the stimulation with IFN-γ, butyrate decreased the level of STAT1 in the nuclear of Flag-STAT1 WT cells (Fig. 2D), supporting the suppression of butyrate on STAT1 activation. The non-acetylated STAT1 mutant at the K410 or K413 diminished the butyrate-induced reduction of STAT1 level in the nuclear (Fig 2D). The luciferase assay showed that butyrate decreased STAT1 activity in HCT116 cells with Flag-STAT1 WT plasmid, while STAT1 mutant at the K410 or K413 (STAT1 null cells overexpressed FLAG-STAT1 K410R or K413R) blocked the butyrate-induced reduced STAT1 activity (Fig 2E).

The above findings lead us to hypothesize that butyrate would determine PD-L1 expression by affecting STAT1 acetylation. To test this hypothesis, the binding of STAT1 with PD-L1 promoter in cells with or without butyrate intervention was subsequently examined by ChIP-qPCR. According to the JASPAR motif analysis (http://jaspar.genereg.net/), the transcriptional bind site of STAT1 is shown in Fig 2F. The data showed that the binding of STAT1 with PD-L1 promoter was significantly decreased by butyrate in Flag-STAT1 WT cells, and this decrease couldn't be observed in FLAG-STAT1 K410R and K413R cells (Fig 2G). These experimental results demonstrated that butyrate impairs STAT1 nuclear translocation to reduce the binding of STAT1 with PD-L1 promoter, thereby suppressing the expression of PD-L1, which is reliant on STAT1 acetylation, in CRC cells.

### Butyrate enhances the cytotoxicity of CD8+ T cells against CRC cells

**Fig. 3:**
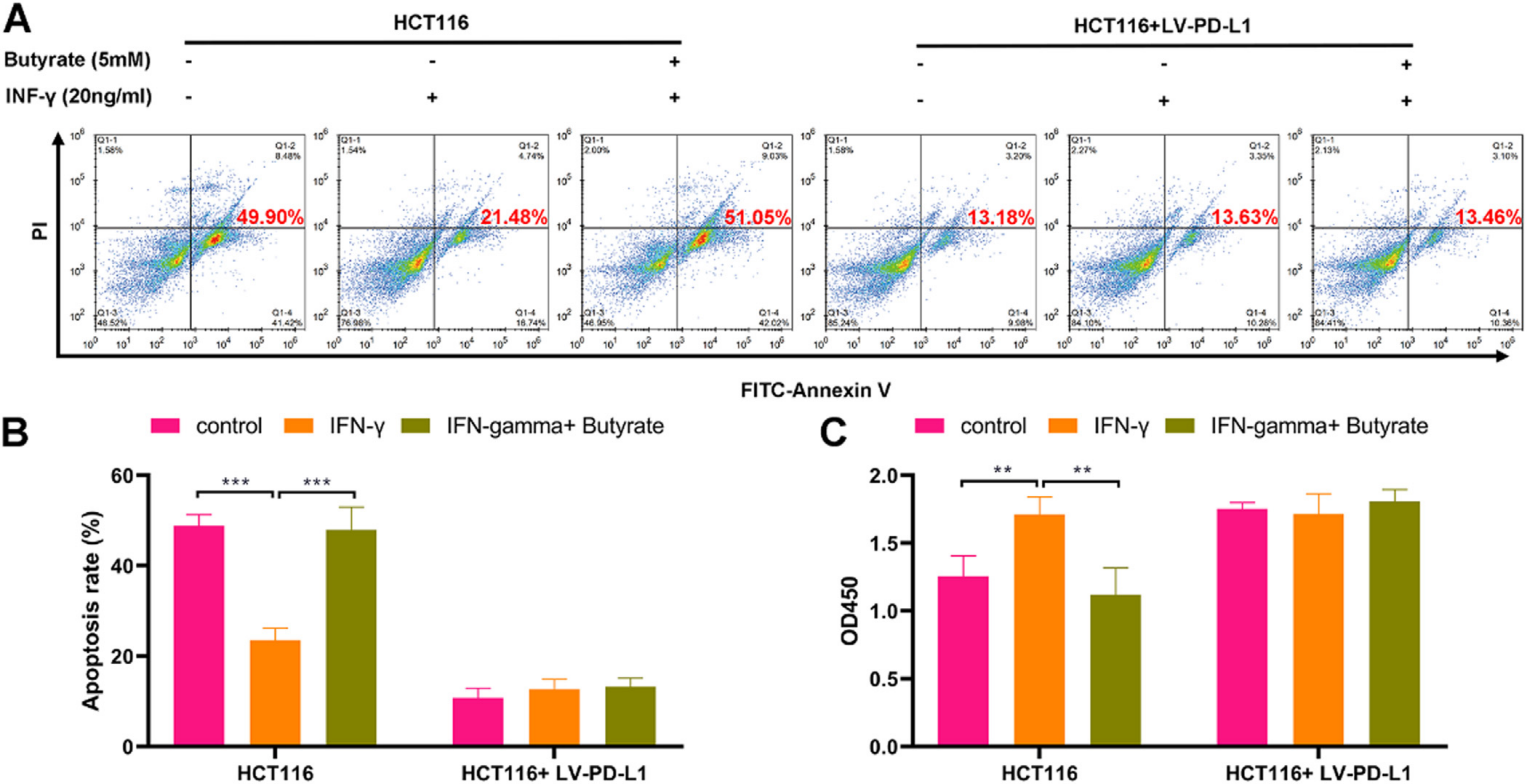
Butyrate enhances cytotoxicity of CD8+ T-cells against CRC cells. Before the treatment of IFN-γ combined with or without butyrate, HCT116 cells were transfected with LV-PD-L1 or the control plasmids. After finishing the treatment, HCT116 cells were co-cultured with TALL-104 cells for 24h. (A) The cell apoptosis detected by flow cytometry. (B) The cell viability detected by CCK-8 assay. ** p < 0.01 and *** p < 0.005.

To determine whether butyrate impacts CD8+ T-cell-mediated killing of CRC cells, HCT116 cells and TALL-104 cells were co-cultured under the treatment with or without IFN-γ combined with or without butyrate. The results showed that the cytotoxicity of TALL-104 cells was weakened in response to the treatment with IFN-γ, as revealed by decreasing amounts of apoptotic HCT116 cells (Fig 3A and B) and increasing cell viability of HCT116 cells (Fig 3C). Moreover, butyrate enhanced TALL-104 cell cytotoxicity that against HCT116 cells under the IFN-γ stimulation (Fig 3A-C). However, the promotive role of butyrate in the cytotoxicity of TALL-104 cells against HCT116 cells was diminished after overexpressing PD-L1 in HCT116 cells (Fig 3A-C). Therefore, butyrate could enhance the cytotoxicity of CD8+ T-cells against CRC cells via PD-L1.

### Butyrate induces anti-tumor immune by enhancing CD8+ T-cell infiltration

**Fig. 4:**
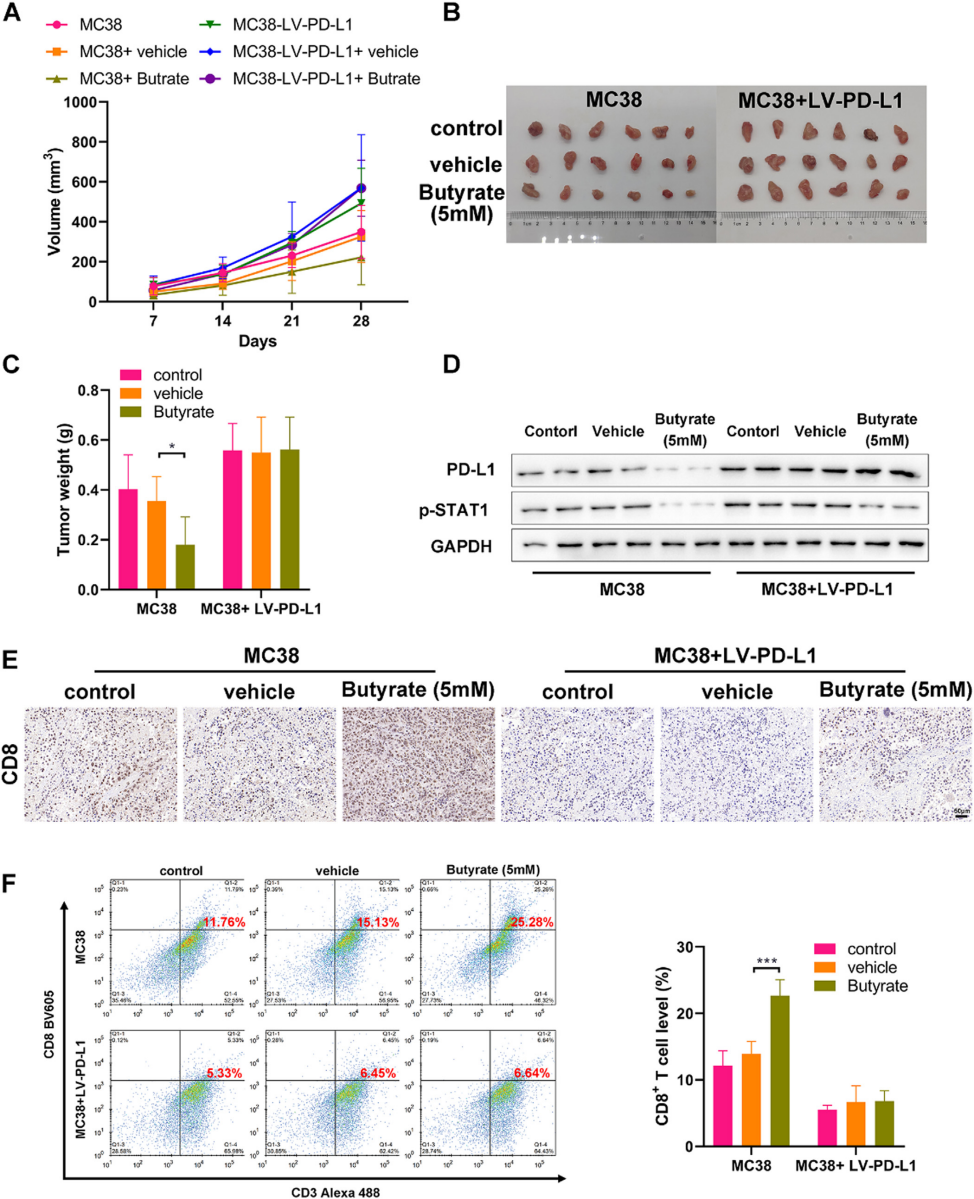
Butyrate induces anti-tumor immune by enhancing CD8+ T-Cell infiltration. CRC mouse model was established with control or PD-L1 overexpressing MC38 cells to evaluate the anti-tumor efficacy of butyrate *in vivo*. (A) The tumor growth curve. (B) The image of collected tumors harvested from each group on day 21. (C) Tumor weight. (D) WB detection of expression of PD-L1 and p-STAT1 in the collected tumors. (E) IHC staining of the collected tumors for CD8. (F) CD8+ T-cell proportion in single cell suspensions of the collected tumors stained for detecting (CD3+ CD8+) by flow cytometry. * p < 0.05 and *** p < 0.005.

Finally, the CRC mouse model was established with control or PD-L1 overexpressing MC38 cells to evaluate the anti-tumor efficacy of butyrate *in vivo*. Starting from the tumor volume reached 50 mm^3^, tumor-bearing mice (n = 6 per group) were treated with saline or sodium butyrate twice a week, and then monitored tumor progression once a week. Tumor growth of mice from the MC38+ Butyrate group was obviously lower than those from the MC38 and MC38+vehicle groups (Fig 4 A‒C). WB analysis on tumors showed that butyrate intervention caused a marked decrease in the expression of PD-L1 and p-STAT1 (Fig 4D), which is consistent with the previous *in vitro* study. However, the *in vivo* anti-tumor activity of butyrate was blocked when the tumor overexpressed PD-L1 ((Fig 4 A‒C). Meanwhile, butyrate intervention also reduced the activation of STAT1 in PD-L1 overexpressing tumors but had no obvious effect on PD-L1 expression (Fig 4D).

To determine whether butyrate could induce anti-tumor immunity, the present study further investigated the activation of tumor-infiltrating CD8+ T-cells. IHC analysis showed that butyrate led to an up-regulation of CD8 expression in the control MC38 tumor, while PD-L1 overexpression could diminish its effect (Fig 4E). Flow cytometry analysis confirmed the result of IHC, showing that butyrate dramatically increased the proportion of CD3+CD8+ T-cells in the tumor tissue in comparison to that in the control (Fig 4F). Notably, it was found that PD-L1 overexpression caused an obvious suppression of tumor-infiltrating CD8+ T-cells, and there was no significant change of the percentage of CD8+ T-cells between diverse treatments in the PD-L1 overexpressing tumor (Fig 4 E and F). These data collectively indicate that butyrate may induce an anti-tumor response by increasing the proportion of CD8+ T-cells in the tumor tissue via suppression of the STAT1/PD-L1 axis.

## Discussion

In the last decade, immune checkpoint blockade has been considered a novel strategy with great promise for several types of advanced cancers.^22^^,^^23^ Since a series of tolerogenic and immunosuppressive mechanisms induced by the tumor, most cancer patients show poor clinical responses to these novel treatments.^24^^,^^25^ Considering the complicated regulation of T-cell functions in the tumor immune microenvironment, there is significant room in exploring approaches to bypass or overcome immune evasion by tumors.^26^

Over the past three decades, butyrate has been reported to exert a potent anti-tumor activity in CRC.^27^^,^^28^ Many studies illustrated that butyrate could induce cell cycle arrest, enhance cell apoptosis, suppress cell proliferation, and impair angiogenesis, thereby inhibiting the malignant activities of CRC cells.^29^, ^30^, ^31^ As known, PD-L1 expression is critical for immune clearance during tumor progression.^32^ Notably, as a HADC inhibitor, butyrate has shown the suppression on IFN-γ-induced STAT1 activation that contributes to PD-L1 expression in a previous publication.^20^ To the authors’ knowledge, as not widely reported, the effect of butyrate on immunotherapies in CRC remains largely unknown.

Recently, high PD-L1 levels have been reported to be correlated with a poor prognosis in CRC patients, which could be regulated by the IFN-γ/STAT1 axis.^17^ A number of recent publications supported that STAT1 signaling is a promising target for regulating the PD-L1 levels in cancer cells.^33^^,^^34^ For example, Zhao et al. recently demonstrated that butein decreased the mRNA level of STAT1 to suppress PD-L1 transcription, thereby achieving tumor immunity regulation.^35^ STAT1 signaling activation is known to require phosphorylation of the C-terminal Ser727. As an additional mode of epigenetic modification of STAT1, STAT1 acetylation could counteract IFN-γ-induced STAT1 phosphorylation, thereby suppressing nuclear translocation and even target gene expression.^36^ In this study, the authors have investigated that butyrate transcriptionally regulates IFN-γ-induced PD-L1 in human CRC cells and uncovered the molecular mechanisms that butyrate suppresses PD-L1 expression through increasing STAT1 acetylation. CD8 T cell cytotoxicity is an important parameter in evaluating the efficacy of the antitumor immune response.^37^ Then, the present study also indicated that these investigations have functional significance in coculture experiments, which showed butyrate intervention in CRC cells was sufficient to strengthen CD8 T cell function via PD-L1.

Infiltration of immune effector cells, including T cells and natural killer cells, into tumors plays a potent role in suppressing tumor growth.^38^^,^^39^ Most significantly, *in vivo* experiments showed that the administration of butyrate led to the suppression of STAT1 signaling, down-regulation of PD-L1, and a decrease in infiltration of CD8+ T-cells that resulted in facilitating immune clearance and tumor growth suppression. This is the first study to clarify the regulation role of butyrate in tumor immunity. In addition, the current study also revealed the specific molecular mechanism of butyrate in upregulating PD-L1 expression via INF-γ–STAT1–PD-L1, which in turn facilitates the immune clearance of CRC and suppresses tumor progression. However, there are several limitations. First, this study lacks an investigation into the role of butyrate in other immune effector cells, which will be a focus of a future study. Besides, the present study remains preliminarily nature while the underlying mechanisms in more depth are required to be verified by further experiments.

In conclusion, the present study identified a novel anti-tumor effect of butyrate, which exerts by suppressing PD-L1 transcription via STAT1 acetylation, providing scientific information to understand the pivotal role of butyrate in tumor immunity.

## Funding

This research did not receive any specific grant from funding agencies in the public, commercial, or not-for-profit sectors.

## CRediT authorship contribution statement

**Yan Zhang:** Conceptualization, Methodology, Validation. **Yuan Tao:** Data curation, Investigation, Resources, Validation. **Yuqing Gu:** Data curation, Writing – original draft. **Qiujie Ma:** Investigation, Methodology, Writing – review & editing.

## Declaration of Competing Interest

The authors declare no conflicts of interest.
